# Impairments in Brain Perfusion, Metabolites, Functional Connectivity, and Cognition in Severe Asymptomatic Carotid Stenosis Patients: An Integrated MRI Study

**DOI:** 10.1155/2017/8738714

**Published:** 2017-02-01

**Authors:** Tao Wang, Feng Xiao, Guangyao Wu, Jian Fang, Zhenmeng Sun, Hongliang Feng, Junjian Zhang, Haibo Xu

**Affiliations:** ^1^Department of Neurology, Zhongnan Hospital of Wuhan University, Wuhan, China; ^2^Department of Radiology, Zhongnan Hospital of Wuhan University, Wuhan, China

## Abstract

Carotid artery stenosis without transient ischemic attack (TIA) or stroke is considered as “asymptomatic.” However, recent studies have demonstrated that these asymptomatic carotid artery stenosis (aCAS) patients had cognitive impairment in tests of executive function, psychomotor speed, and memory, indicating that “asymptomatic” carotid stenosis may not be truly asymptomatic. In this study, when 19 aCAS patients compared with 24 healthy controls, aCAS patients showed significantly poorer performance on global cognition, memory, and executive function. By utilizing an integrated MRI including pulsed arterial spin labeling (pASL) MRI, Proton MR Spectroscopy (MRS), and resting-state functional MRI (R-fMRI), we also found that aCAS patients suffered decreased cerebral blood flow (CBF) mainly in the Left Frontal Gyrus and had decreased NAA/Cr ratio in the left hippocampus and decreased connectivity to the posterior cingulate cortex (PCC) in the anterior part of default mode network (DMN).

## 1. Introduction

Carotid artery stenosis without transient ischemic attack (TIA) or stroke is considered as “asymptomatic” [1]. However, recent studies have demonstrated that these asymptomatic carotid artery stenosis (aCAS) patients had cognitive impairment in tests of executive function, psychomotor speed, and memory [2–4]. However, the pathophysiological mechanism of cognition impairment in aCAS patients has not been understood thoroughly.

In the past few years, several imaging techniques have increasingly been used to study cognition impairment in humans, such as pulsed arterial spin labeling (pASL) MRI, Proton MR Spectroscopy (MRS), and resting-state functional MRI (R-fMRI) [5–7]. pASL MRI can be used to detect regional cerebral blood flow (CBF) while MRS can measure relative changes in metabolites, which had been changed at a very early stage of cognitive impairment. R-fMRI evaluates the temporal correlation between the spontaneous blood oxygenation level-dependent (BOLD) fluctuations at resting-state [8]. We tested two parameters, functional connectivity (FC) and amplitude of low-frequency fluctuation (ALFF). FC reflects interregional cooperation while ALFF refers to the intensity of regional brain activity [9, 10]. Since lots of previous studies have reported that the posterior cingulate cortex (PCC) is one of the key nodes of default mode network (DMN) and cognition, the region of interest (ROI) in this study was set at this node [11, 12].

In this study, our first goal was to detect the differences in CBF, metabolites, and intrinsic functional network connectivity between aCAS patients and healthy controls. Secondly, we tried to find whether these differences were related to the cognition differences.

## 2. Methods

### 2.1. Participants, Inclusion Criteria, and Exclusion Criteria

We recruited testable aCAS patients from Zhongnan Hospital affiliated to Wuhan University between January 2015 and June 2016. The inclusion criteria include the following: (1) age from 55 to 80 years; (2) ICA stenotic degree *⩾* 70%; (3) right-hand dominance; (4) being free of stroke, TIA, dementia, or depression; (5) Modified Rankin Scale: score 0 or 1; and (6) no major psychiatric disease or other medical conditions. The exclusion criteria were (1) contralateral internal carotid artery stenosis *⩾* 50%; (2) posterior circulation diseases; (3) MMSE < 26; (4) functional disability (Modified Rankin Scale ≥ 2); (5) severe systemic diseases and neuropsychiatric diseases (such as congestive heart failure and history of stroke); (6) any contraindications for MR scan (e.g., metal implants); and (7) low education level (<6 years). In the meanwhile, we also recruited 24 age and education level matched healthy controls. Written informed consent was obtained from all participants. All the study procedures were approved by the Zhongnan Hospital Review Board.

### 2.2. Cognition Assessments

Cognition assessments were performed within 7 days of MRI scan. The MMSE and MoCA Beijing Version were utilized to assess the global cognition. The Digit Symbol Test required subjects to translate numbers to symbols in a given time and correct translations within 90 seconds were recorded. The Rey Auditory Verbal Learning Test (RAVLT) was applied to evaluate the memory and verbal learning ability. The participant should try to recall the words as much as he/she can remember. This procedure was repeated five times and then followed a delayed recall after thirty minutes. The total number of the words immediately recalled during the first five repeats and the sum of the delayed recall were record accordingly. In the Verbal Memory Test, participants were required to repeat orally presented lists of numbers, beginning with a 2 number sequence, and each correct performance was followed by 1 additional number. In the forward span, participants were asked to retell the span in forward order. In the backward span, participants were asked to retell the span in reverse order.

### 2.3. Brain Imaging Collection

MRI images were acquired using a 3.0 Tesla Siemens scanner at Zhongnan Hospital. pASL perfusion images were collected using Q2TIPS II technique. Scan parameters were TR = 2500 ms, TE = 11 ms, FOV = 240 × 240 mm^2^, matrix = 64 × 64, FA = 90°, and slice thickness = 6 mm. ^1^H MRS chemical shift imaging (CSI) was conducted according to the following protocol: TR = 1600 ms, TE = 135 ms, FOV = 160 × 160 mm^2^, matrix = 16 × 16, and voxel size = 10 × 10 × 16 mm^3^. R-fMRI were acquired using EPI sequence: Repetition Time = 2000 ms, Echo Time = 30 ms, Flip Angle = 90°, number of slices: 33, slice thickness: 3.8 mm, gap: 1 mm, data matrix: 64 × 64, and Field of View = 240 × 240 mm^2^.

### 2.4. Image Processing

#### 2.4.1. pASL

relCBF were automatically generated by Siemens workstation and then were normalized to EPI template provided by Statistical Parametric Mapping 8 (SPM8). The final voxel size was 3 × 3 × 3 mm^3^. Each subject's relCBF map was transformed into standard MNI space using these transformation parameters. These normalized relCBF maps were then smoothed with 8 mm FWHM isotropic Gaussian kernel. SPM8 were then used to identify significant different regions between two groups.

#### 2.4.2. MRS

Since previous study had demonstrated that abnormal level of hippocampus metabolites may mediate cognitive performance, we selected 4–6 voxels from both hippocampi using workstation Spectroscopy software [13]. Then the concentrations of* N*-acetyl-aspartate (2.02 ppm), choline (3.22 ppm), creatine (3.02 ppm), the ratio of NAA/Cr, and the ratio of Cho/Cr were measured in each selected voxel.

#### 2.4.3. R-fMRI Preprocessing

R-fMRI preprocessing was performed with Data Processing Assistant for resting-state fMRI (DPABI 2.1). The first 10 volumes were abandoned. Then, the images were corrected for slice timing and realigned. (Subjects with a maximum displacement in the *x*, *y*, or *z* direction of more than 1 mm or more than 1° of angular rotation about any axis for any of the 230 volumes were excluded from this study. No subject was excluded according to this criterion.) Afterward, images were normalized into standard MNI space and smoothed with 8 mm FWHM isotropic Gaussian kernel.

#### 2.4.4. ALFF

ALFF calculation was performed with resting-state fMRI Data Analysis Toolkit (REST 1.8). One-sample* t*-test was performed using SPM8 in each group to detect the regions with higher-than-mean ALFF. These mALFF images were then performed for two-sample* t*-test to determine between-group differences. Significant different regions were shown on MNI templates. The two-sample* t*-test results were restricted within the mask made from the results of one-sample* t*-tests performed for two groups.

#### 2.4.5. Functional Connectivity

All images were filtered with a 0.01–0.08 Hz band-pass filter to reduce the noise before FC analysis. The ROI was located in the bilateral PCC (centered MNI coordinates: 0, −56, 25, *r* = 10 mm) [14]. The mean ROI signal was counted by averaging all voxels in bilateral PCC. The ROI time course was used to perform correlation analysis with all other voxels in the brain. To normalize the correlation coefficients, Fisher* z*-transform was then applied. One-sample* t*-test was performed using SPM8 in each group to detect the regions with significant connectivity to the PCC. These* z*-FC images were then performed for two-sample* t*-test to determine between-group differences. Significant different regions were shown on MNI templates. The two-sample* t*-test results were restricted within the mask made from the results of one-sample* t*-tests performed for two groups.

### 2.5. Statistical Analysis

We used IBM SPSS 20.0 and SPM8 to perform statistical analyses. Continuous variables were assessed with Mann–Whitney test or two-sample* t*-test. Categorical variables were assessed with Chi-squared or Fisher exact test if the expected number was ≤5. Significance was defined as *P* < 0.05. Education and age were defined as covariates in all tests involving cognition. After the analysis of pASL, ALFF, and FC, regions with significant differences between two groups were defined as ROIs; then Spearman analysis was performed to detect the relationship between these MRI differences and cognition scores.

## 3. Results

### 3.1. Patient Characteristics and Neuropsychological Evaluation

We enrolled 19 aCAS patients and 24 healthy controls. No significant difference was found in educational years, gender ratio, age, or vascular risk factors. Compared with the controls, aCAS patients had significantly poorer performances on global cognition (represented by MMSE and MoCA), memory (represented by Verbal Memory Test and Rey Auditory Verbal Learning Test), and executive function (represented by Digit Symbol Test) (Table 1).

### 3.2. Difference in CBF between Two Groups

**Figure 1:**
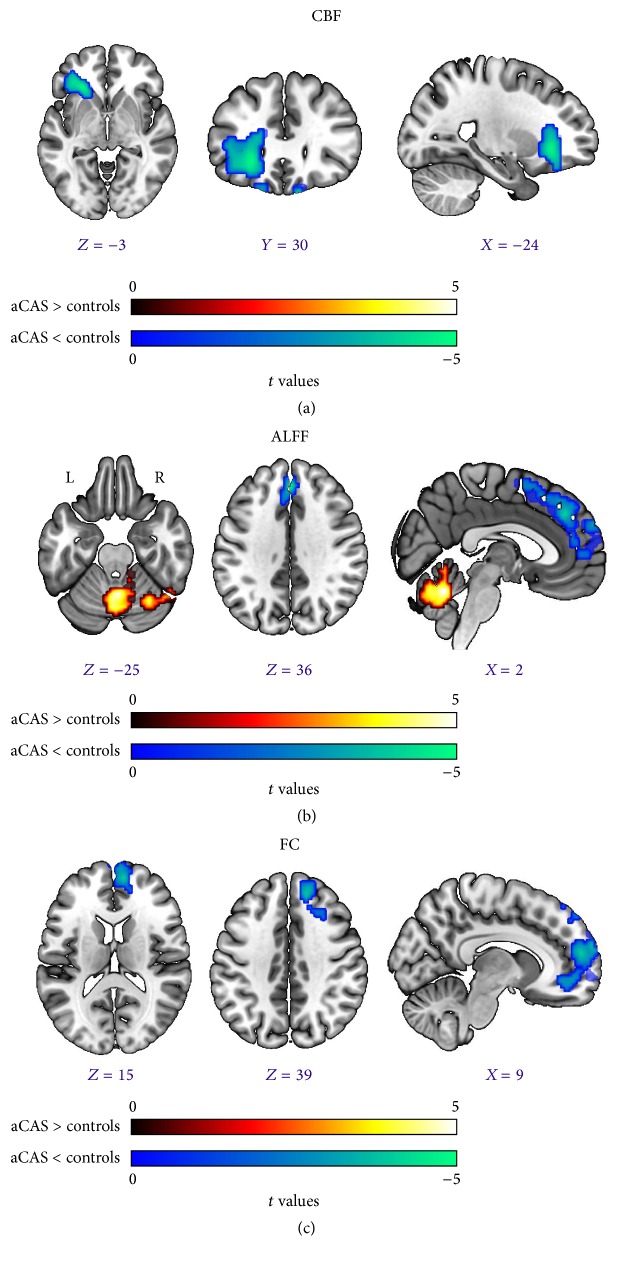
CBF, ALFF, and FC differences between two groups. (a) CBF differences between two groups. (b) Differences of ALFF between two groups. (c) FC differences between aCAS patients and controls. The color bar in (a), (b), and (c) represents *t* values.

aCAS patients showed decreased CBF in the Left Inferior Frontal Gyrus when compared with healthy controls (Table 2, Figure 1(a)). The result was corrected using the AlphaSim program, with a setting at *P* < 0.01 and number of voxels > 489, which corresponded to a corrected *P* < 0.025.

### 3.3. Difference in MRS Finding between Two Groups

In the study of MRS in both hippocampi, we only found that the NAA/Cr in the left hippocampus had significant difference between two groups (Table 3).

### 3.4. Significant ALFF Differences between Two Groups

Significantly decreased ALFF in the Left and Right Supra Medial Frontal Lobes were found in aCAS patients. The aCAS patients also showed increased ALFF in Cerebellum (Table 4 and Figure 1(b)). The result was corrected using the AlphaSim program, with a setting at *P* < 0.01 and number of voxels > 277, which corresponded to a corrected *P* < 0.01.

### 3.5. Differences of FC to PCC between aCAS Patients and Healthy Controls

Compared with controls, the aCAS patients showed decreased connectivity to the PCC mainly in the Right Supra and Medial Frontal Gyrus. No region showing increased FC to the PCC was found (Table 5 and Figure 1(c)). The result was corrected using the AlphaSim program, with a setting at *P* < 0.01 and number of voxels > 203, which corresponded to a corrected *P* < 0.025.

### 3.6. Relationship between Imaging Findings and Cognition Scores

No significant correlation between MRI findings and cognition scores was found (*P* > 0.05 for all).

## 4. Discussion

Consistent with previous studies [2, 4], we found that aCAS patients had significantly poorer performances on global cognition (represented by MMSE and MoCA), memory (represented by Verbal Memory Test and Rey Auditory Verbal Learning Test), and executive function (represented by Digit Symbol Test) when compared with the controls. Therefore, aCAS patients should be no longer considered as “asymptomatic.” Results from the integrated MRI study could mainly be divided into three parts. Firstly, aCAS patients suffered from decreased CBF mainly in the Left Frontal Lobe when compared with healthy controls. Secondly, aCAS patients had lower NAA/Cr ratio in the left hippocampus. Thirdly, aCAS patients had both decreased ALFF and decreased connectivity to the PCC in the Right Supra and Medial Frontal Gyrus.

This study also showed the adverse effect of decreased cerebral blood flow on cognition. Cerebral hypoperfusion is one of the most important pathophysiological mechanisms of vascular cognitive impairment in vascular diseases [15, 16]. Abnormality of neuron electric activities and protein synthesis can partly explain the correlation between cerebral hypoperfusion and cognition impairment. What is more, reduced CBF was mainly located in the Left Frontal Lobe, which consists of key regions that mediate cognitive function.

NAA is an exclusive amino acid in neuron, and the level of NAA can reflect the neuronal viability [17]. The level of Cho has been found to be related to membrane synthesis and degeneration [18]. Abnormality of these signals had been demonstrated in a number of central nervous system diseases [19, 20]. Similar to our previous study [13], we found that aCAS patients had lower NAA/Cr ratio in the left hippocampus.

In recent years, an increasing number of studies have demonstrated that cognitive function performance are not dependent on individual brain region, but dependent on regions, thereby forming networks [21]. A number of studies have already demonstrated this in patients with neurodegenerative diseases [22, 23]. Among these networks, the default mode network (DMN) has been increasing as noticed in recent years [24]. As demonstrated by functional MRI and PET studies, the most common DMN components are the posterior cingulate cortex, the medial prefrontal cortex, the anterior cingulate cortex, the inferior parietal lobule, and other regions [11, 14]. Although the precise functions of DMN remain largely unclear, studies have shown that it plays an important role in cognition and self-monitoring [25]. Another reason why we selected DMN for analysis was that previous studies have demonstrated that DMN is especially easily affected by hypoperfusion [26].

Of the indexes utilized in R-fMRI, ALFF is a useful index to reflect spontaneous neuronal activity [27–30]. We found that aCAS patients showed decreased ALFF in the Left and Right Supra Medial Frontal Gyrus when compared with healthy controls, which belong to the anterior part of DMN. Since the anterior part of DMN is especially associated with executive function, the cognitive disturbances of aCAS patients could be partly due to the decreased activities of these regions [31–33]. Increased ALFF in the Cerebellum was also found in aCAS patients, and we speculate that the increased ALFF in these regions might reflect compensation due to the low perfusion damage.

We also compared the FC to the PCC between two groups and found that the aCAS patients showed decreased connectivity mainly in the Right Supra and Medial Frontal Gyrus when compared with healthy controls. There was no region that showed increased connectivity to the PCC. The decreased regions were also overlapped with the anterior part of the DMN. Therefore, the cognitive impairment could also be partially attributed to the decreased FC to the PCC in the anterior part of DMN, although no significant correlation was found.

Our study had limitations that required discussion. Firstly, the sample size was relatively small. Secondly, the cerebral hemisphere of the stenosis side often exhibits greater brain atrophy. In that situation, the proportion of cerebrospinal fluid occupying the ROI for metabolites measurement is higher, thereby reducing the accuracy [34]. Thirdly, the cerebral metabolism was obtained using multivoxel method, while single-voxel method could provide more accurate measurement [35]. In addition, future studies should also examine the influence of silent infarcts and microbleeds on cognition, as they also affect CBF and subsequent neurocognitive outcomes.

In summary, the aCAS patients showed significantly poorer performance on global cognition, memory, and executive function. By utilizing this integrated MRI, we also found that aCAS patients suffered decreased CBF mainly in the Left Frontal Gyrus and had decreased NAA/Cr ratio in the left hippocampus and decreased connectivity to the PCC in the anterior part of DMN.

## Figures and Tables

**Table 1:** Basic demographics and cognitive test scores of enrolled subjects.

Characteristics	Patients (*n* = 19)	Controls (*n* = 24)	*P* value
Age (years)	68.0 ± 5.6	64.5 ± 7.3	0.08
Male : female	15 : 4	19 : 5	1.00
Education (years)	9.9 ± 3.3	10.9 ± 3.4	0.21
Hypertension	19	18	0.70
Diabetes mellitus	4	4	1.00
Hypercholesterolemia	13	12	0.64
Stenotic side			
Left	7	N/A	
Right	12	N/A	
MMSE	26.8 ± 0.7	27.4 ± 0.7	0.02
MoCA	23.3 ± 1.2	24.2 ± 1.6	0.02
Verbal memory test			
Forward digit span	5.8 ± 1.0	6.5 ± 0.9	0.04
Backward digit span	3.8 ± 0.8	4.5 ± 0.8	0.02
Rey Auditory Verbal Learning test			
Immediate recall	31.0 ± 4.5	35.8 ± 5.6	<0.01
Delayed recall	4.6 ± 1.6	6.5 ± 1.1	<0.01
Digit Symbol Test	28.0 ± 4.7	31.5 ± 5.5	0.03

**Table 2:** CBF difference between two groups and their location.

	Number of voxels	Peak MNI coordinate			Peak MNI coordinate region	Peak *T* value
		*X*	*Y*	*Z*		
1	603	−24	30	−3	Left Inferior Frontal Gyrus, Brodmann area 11	−3.91

**Table 3:** Significant NAA/Cr difference in the left hippocampus between two groups.

	aCAS	Controls	*P* value
NAA/Cr	1.6 ± 0.1	1.7 ± 0.1	0.02

**Table 4:** Significant ALFF differences between two groups with their location.

	Number of voxels	Peak MNI coordinate			Peak MNI coordinate region	Peak *T* value
		*X*	*Y*	*Z*		
1	708	0	−57	−24	Cerebellum	5.23
2	356	0	39	36	Left and Right Supra Medial Frontal Lobes	−6.67

**Table 5:** Significant connectivity differences to the PCC between two groups with their location.

	Number of voxels	Peak MNI coordinate			Peak MNI coordinate region	Peak *T* value
		*X*	*Y*	*Z*		
1	286	9	57	15	Frontal_Medial_R	−4.20
2	220	15	42	39	Frontal_Sup_R	−4.00
